# Identification of gametocyte-associated *pir* genes in the rodent malaria parasite, *Plasmodium chabaudi chabaudi* AS

**DOI:** 10.1186/s13104-023-06322-1

**Published:** 2023-04-19

**Authors:** Deirdre A. Cunningham, Adam J. Reid, Caroline Hosking, Katrien Deroost, Irene Tumwine-Downey, Mandy Sanders, Jean Langhorne

**Affiliations:** 1 The Francis Crick Institute, London, UK; 2 Wellcome Sanger Institute, Cambridge, UK; 3 Present Address: Wellcome/Cancer Research UK Gurdon Institute, University of Cambridge, Cambridge, CB2 1QN UK

**Keywords:** Multigene family, *Pir*, Transcriptome, Gametocyte, *Plasmodium chabaudi*

## Abstract

**Objective:**

To analyse the transcriptional profiles of the *pir* multigene family of *Plasmodium chabaudi chabaudi* in male and female gametocytes isolated from the blood of infected mice.

**Results:**

Infected red blood cells containing female and male *P. chabaudi* gametocytes transcribe a distinct set of genes encoded by the multigene family *pir*. The overall patterns are similar to what has been observed in the close relative *P. berghei*, but here we show that gametocyte-associated *pir* genes are distinct from those involved in chronic blood-stage infection and highlight a male-associated *pir* gene which should be the focus of future studies.

**Supplementary Information:**

The online version contains supplementary material available at 10.1186/s13104-023-06322-1.

## Introduction

The genomes of *Plasmodium* species contain several multigene families, the largest of which is the *pir* multigene family shared by *Plasmodium vivax* which infects humans*, Plasmodium* species infecting monkey and apes and members of the *Vinckei* subgenus infecting rodents (reviewed by [1]). The role of *pir* genes is best understood in the rodent-infecting parasite *P. chabaudi*. We have shown that clusters of *pir* genes, named ChAPLs, play a role in establishing chronic infection during the asexual part of the life cycle [2]. We have also shown that the L1 *pir* genes, which are enriched in these clusters, are associated with higher virulence [3, 4]. We have not previously examined the expression of *P. chabaudi pir* genes in other parts of the life cycle. There have however been comprehensive analyses of *pir* gene expression across the complete life cycle of another rodent-infecting parasite, *P. berghei.* Using both bulk and single-cell RNA-seq [5, 6], these have shown that *pir* genes are highly expressed not only in the asexual blood forms, but in the sexual gametocyte stages, which develop from the asexual blood stages. These are the parasite forms transmitted to mosquitoes and which are the subject of intense study to develop transmission blocking therapies. While we have shown that the role of *pir* genes in asexual stages may be conserved in *P. berghei*, there was not a clear association between genomic clusters of genes and chronic infection as we saw for *P. chabaudi* [2]. Here we aim to fill this gap and to determine whether there is any relationship between the *pir* genes involved in chronic infection and those involved in gametocyte biology, or whether these represent distinct subsets of *pir* genes.

Here we have analysed transcription of *pir* genes in *Plasmodium chabaudi chabaudi* (AS) gametocytes during a blood-stage infection in C57BL/6 mice. Using transgenic parasites, in which male and female gametocytes are specifically tagged with a fluorochrome via Dynein (male) [7] or CCp2 (female) [7], we were able to isolate male and female gametocytes and compared transcriptomes of these parasites to the transcriptome of asexual stage parasites. Overall *pir* transcription is lower in male and female gametocytes than in asexual stage parasites, and while the long L1 forms predominate in asexual parasites, an L4 *pir* is dominant in male gametocytes with the female gametocyte *pir* transcriptome being comprised mainly of short S7s and the ancestral *pir* [8]. This transcriptome data for purified male and female *P. chabaudi* gametocytes supplements the previously published whole transcriptome data for the asexual stages of this model organism [2, 3, 5] and provides a useful resource to the *Plasmodium* community.

## Methods

**Fig. 1:**
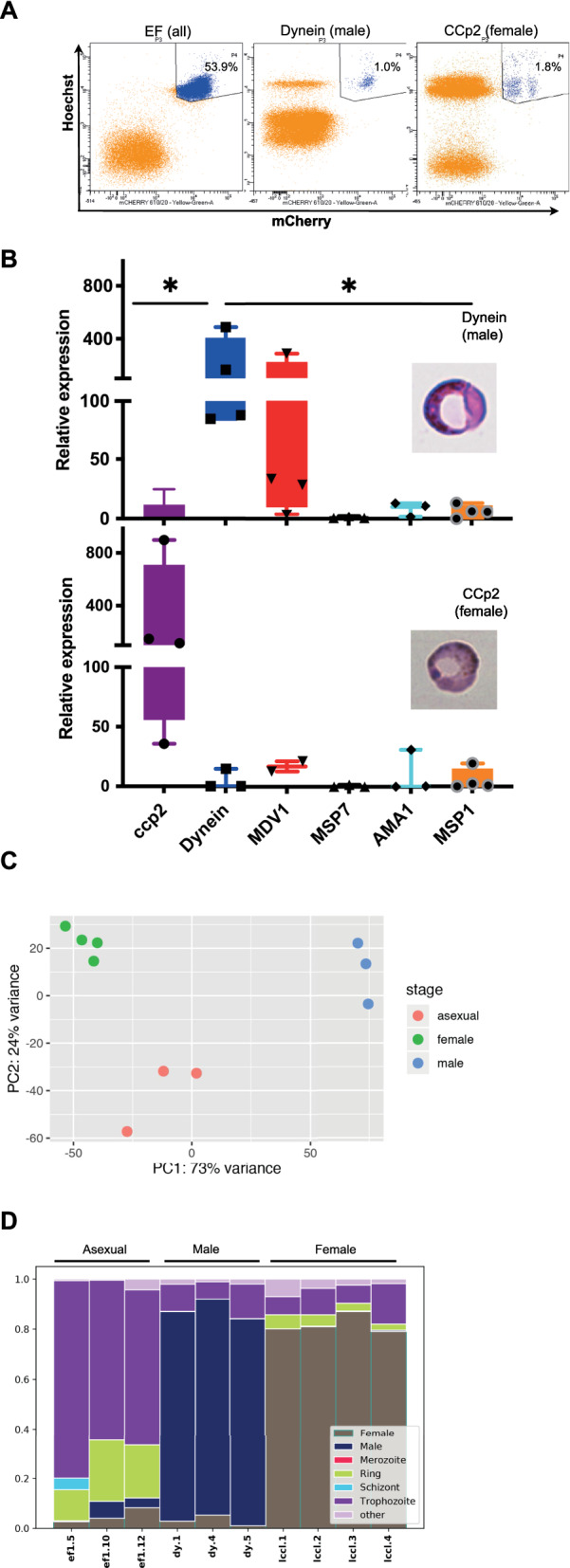
Separation of male and female gametocytes from asexual stage parasites. **A** Infected red blood cells were sorted on the mCherry tag on a BD FACS Aria Fusion B flow cytometer, d14 post-infection. Nuclei were stained with Hoechst 33342 and mCherry was on male gametocytes (*Pc*ASDHCmC_230p_; n = 3); female gametocytes (PcASCCp2mC_230p_; n = 4) or all infected red blood cells (*Pc*ASEFmC_230p_; n = 3) and the mCherry/ Hoechst 33342 double positive population (P4) collected for RNA extraction and microscopy (representative image). **B** The sorted population (P4) was analysed by QRTPCR using primer sets designed to housekeeping genes and to sexual and asexual stage genes (*Mann–Whitney* *p < *0.05)* and visualised on Giemsa-stained thin blood films. **C** Principal Components Analysis plots of transcriptomes from *P chabaudi* AS male and female gametocytes and asexual stage parasites sorted on mCherry on a BDFACS Aria^™^ Fusion B flow cytometer. **D** Composition of the transcriptomes of each sample with respect to genes annotated to parasite developmental stages

**Fig. 2:**
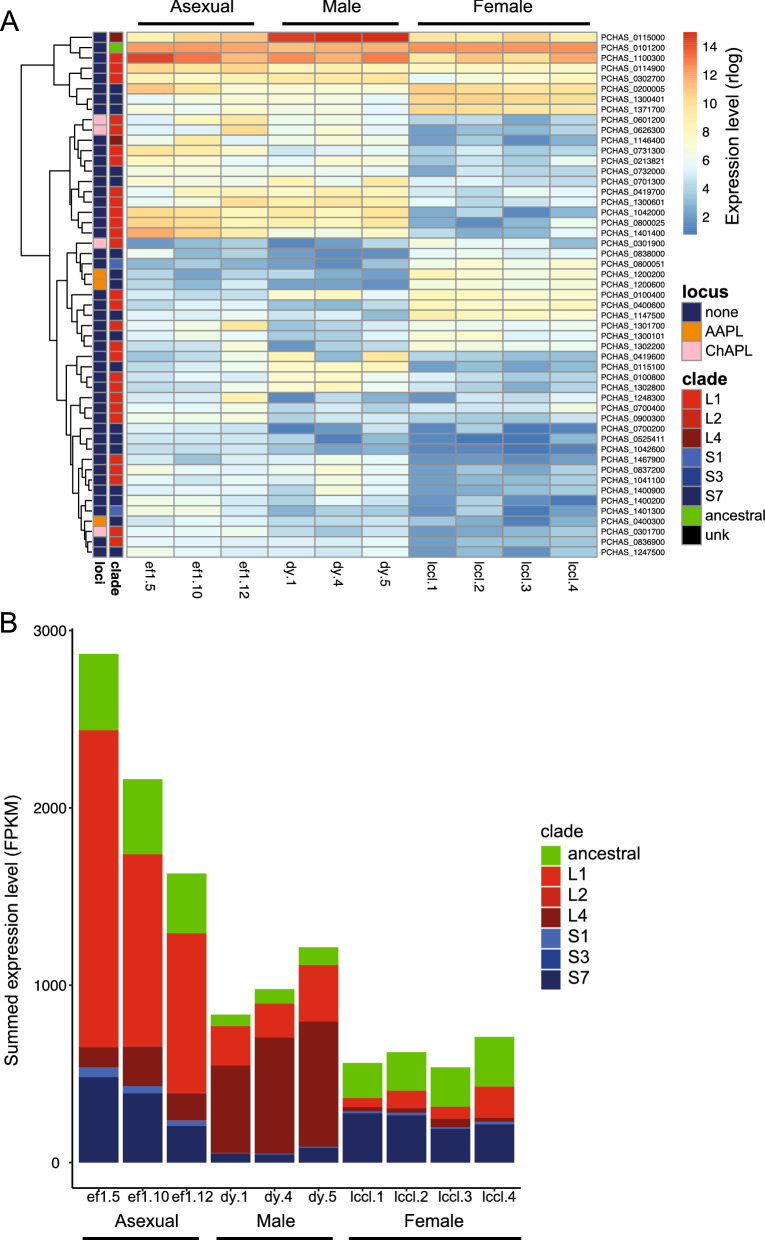
*Pir* gene expression in asexuals, male and female gametocytes. **A** All differentially expressed *pir* genes are shown clustered in a heatmap. Gene expression levels are presented as rlogs. *Pir* genes are classified by locus: ChAPL (Chronicity-Associated Pir Locus), AAPL (Acute-Associated Pir Locus) or none as defined in Brugat et al. [2] and by clade as defined in Otto et al. [7]. This indicates distinctive *pir* gene transcripts present in the male and female gametocyte transcriptomes. **B** The summed expression levels of *pir* genes (now the linear FPKM measurement of gene expression for comparison with Little et al. (5)) in each sample are shown, split into the different clades as in the above panel. This indicates a lower overlap level of *pir* expression in gametocytes with distinct clade differences between male and female gametocytes

For the generation of specifically-tagged male and female gametocyte lines, promoters were chosen based on the published transcriptome for *P berghei* ANKA [7]. A region of approximately 1 kb upstream of the translation start site of the dynein heavy chain gene or of the CCp2 gene was selected, synthesized commercially (Genewiz), digested with AflII and BamH1 and used to replace the *eef1a* promoter to generate the plasmids *Pc*DHC_230p_mC, and *Pc*CCp2_230p_mC (see Additional File 1 for sequences and Additional file 2 for schematic). These genes have been reported to be active specifically in male or female gametocytes [7, 9]. Transgenic parasites were generated by transfection with these plasmids [10] and cloned by serial dilution. Insertion into the targeted 230*p* genomic locus was verified by PCR, as described previously [2] (see Additional file 2 for insertion verification). C57BL/6 mice (n = 5), 6–8 weeks old, were infected with each of the transgenic lines and infected erythrocytes were harvested from those infected mice with a gametocyte density of > 0.1% (3 samples for the Dynein and EF groups, 4 samples for the LCCLccp2 group), at a time when gametocyte production is at a maximum (d14 post-infection) [11]. Mice were housed under reverse light conditions where gametocytes are formed at 0:00 h and maturation into schizonts occurs at 12:00 and blood harvested 2 h before schizogony, a time when we routinely obtain successful transmission to mosquitoes. The mCherry/ Hoechst 33342 infected erythrocytes were separated by sorting on mCherry/ Hoechst 33342 on a BD FACS Aria Fusion B flow cytometer (Fig. 1A and see Additional file 2 for representative gating strategy) and the specificity of the tag for the target population was verified by qRT-PCR using primer sets designed to housekeeping genes and to sexual and asexual stage genes (see Additional file 1 for primer sequences and qRTPCR data), on the sorted mCherry/ Hoechst 33342 positive population (P4; Fig. 1B). Relative expression of the *dynein* and *mdv1* genes was high in the male gametocyte line, compared to the constitutive EF line, with little or no expression of the female *ccp2* gene or the asexual-associated genes *msp7*, *ama1* and *msp1* while conversely, for the female gametocyte line, expression of *ccp2* was high, and *dynein* and asexual genes were low. Statistical analysis (Mann Whitney two tailed non parametric test) of the relative expression of each of the sample groups for each of the genes showed that for the male tagged and sorted samples *p* = *0.029* for Dynein versus ccp2 and for Dynein versus MSP1; for Dynein versus the other asexual associated (MSP7, AMA1) genes *p* = *0.057*; for the female tagged and sorted samples *p* = *0.057* for ccp2 versus Dynein, MSP7, AMA1 and *p* = *0.029* for MSP 1. For MDV1 in both sets of samples *p* > *0.1*, as this gene is highly expressed in both male and female gametocytes. The high *p* values for the female samples are due to one sample being more of an outlier. Global gene expression analysis was performed on the sorted cells. Read counts were normalized using DESeq2 v1.36.0 with default parameters [12]. Principal Components Analysis (PCA) showed 3 distinct populations of female, male and asexual parasite samples as expected (Fig. 1C). To further validate the specificity of the Dynein and Ccp2 promoters we deconvoluted the bulk RNA-seq transcriptomes using scRNA-seq data from the Malaria Cell Atlas using the approach described in Aunin et al. [6, 12](Fig. 1D). This suggested that the male and female gametocyte samples were largely composed of the expected cell types and that the asexual samples were composed of largely trophozoites with a small proportion of ring stages. DESeq2 was used to determine genes differentially expressed (DE) between males, females and asexuals in a pairwise fashion (adjusted p-value < 0.01, log2 fold change > 1 or < − 1) and to calculate rlog normalized expression values for plotting. The *pir* genes DE in any pairwise comparison were used in Fig. 2A. Reads Per Kilobase per Million mapped reads (RPKM) expression values were used in Fig. 2B to make comparison with the work of Little et al. [5] easier. *Pir* gene annotations were derived from Brugat et al. [2].

## Results

The *P. chabaudi* genome contains 210 *pir* genes [2]. In rodent-infecting *Plasmodium* species these comprise long (L) and short (S) forms*,* classified phylogenetically into clades (L1-L4; S1-S8) [7]. In *P. chabaudi* asexual stage parasites, *pir* genes at particular genomic loci associate with either acute or chronic infections (Acute Associated *pir* Locus or AAPL; Chronic Associated *pir* Locus or CHAPL) [2]. While AAPLs are comprised of mainly S7 and some L4 pirs, ChAPLs are comprised of mainly L1 pirs with some L4 pirs. In *P. chabaudi* blood-stage infections initiated by injection of 10^5^ infected red blood cells, we find that there are similarities in *pir* gene expression between asexual, male and female gametocytes, but also groups of genes which are associated specifically with males or females (Fig. 2A). As expected from serially blood-passaged parasites, the predominant *pir* gene in asexual stages is PCHAS_1100300 [3], with the ancestral *pir* also highly expressed [5]. Approximately 25% of *pirs* are differentially expressed between male and female gametocytes and asexual stage parasites (Fig. 2A), and the *pir* transcriptome falls into four groups (i) those common to all, (ii) those shared between male gametocytes and asexual stages, (iii) those specific for female-gametocytes and (iv) those with low expression in female gametocytes, low to medium expression in asexual stages and a range of expression in male gametocytes. Most of the differentially expressed *pirs* were assigned to unclassified loci, with the exception of 2 genes from AAPLs in the female group and 2 from CHAPLs in the male/asexual group (Fig. 2A). The lack of association with AAPL and CHAPL loci is unsurprising these loci were identified through their association with acute and chronic infections occurring post mosquito-transmission [2]. Figure 2B shows that the *pir* transcriptome of male gametocytes is dominated by L4 *pir* genes, in fact this relates to just one gene: PCHAS_0115000. There are also several L1 *pir* genes, expressed more highly in males versus females and asexuals. These are L1s from neither ChAPLs or AAPLs (PCHAS_0100800, PCHAS_0419600 and PCHAS_1302800). As seen in *P. berghei*, the *pir* transcriptome of females is less abundant than in males, although not to the same extent. It is typified more by S7 *pir* genes, two of them from AAPLs (PCHAS_1200200, PCHAS_1200600), but also two L1s, one from a ChAPL (PCHAS_0301900). Asexual parasites are typified by L1s, which are the more abundant subfamily in the genome (82 L1s, 70 S7s, 34 L4s, 16 S1s). It is clear that each parasite form has a distinctive pattern of *pir* transcripts. These parasite forms are transcriptionally very different overall and so this is not surprising per se. More notable are the similarities with the close relative *P. berghei* at those same stages. Single-cell RNA-seq analysis of *P. berghei* showed that males express several S1 and S4 *pir* genes more highly than females, which do not express any *pirs* more highly than males. Variable expression of S1 *pir* genes indicated there may be differences between individual male parasites [13]. Furthermore, the male-specific *pir* genes have common promoter sequences suggesting they may be under control of a particular transcription factor [6]. The relative abundance of *pir* transcripts in males versus females was underscored by analysis of more deeply sequenced bulk transcriptomes [5]. The genomic *pir* repertoires are quite different in these species, despite the rest of the genomes being very similar. Most obviously, L *pir* genes are the most common in *P. chabaudi* and S forms the most common in *P. berghei*. Despite this, we observe relative increases in expression of S *pir* genes in male gametocytes compared to asexual forms. Male forms have higher expression of *pir* genes overall than females, although to a lesser extent than in *P. berghei* [5]. The high expression of one particular L4 *pir* gene in males is of particular interest for understanding the potential role of these genes in parasite transmission and fertilisation. The *pir* genes associated with gametocytes versus asexuals were sometimes from AAPLs and ChAPLs in females, but never in males. The highly expressed L4 in male gametocytes represents the best target for a gametocyte-specific role of *pir* genes.

## Conclusions

The ancestral *pir* gene, PCHAS_0101200, is highly expressed in male and female gametocytes and in asexual stage parasites. The L1 *pir*, PCHAS_1100300, dominant in the transcriptome of serially blood passaged asexual stage parasites [3] also scores very highly in male and female gametocytes. Male forms have higher expression of *pir* genes overall than females, as observed in other species of rodent malaria parasites, however the difference is less pronounced than in *P. berghei*. L4 *pirs* form the highest proportion of the male gametocyte transcriptome, due to the high expression of a single L4 *pir* gene, PCHAS_0115000, the highest scoring *pir* of the male transcriptome. The role of this *pir* in males warrants further investigation.

### Limitations

1. Only a single time point in the development of female and male gametocytes in the blood of infected C57BL/6 mice was investigated.
2. Flow cytometry for the separation of biological populations is not 100% clean, hence multiple tests were applied to support specificity.
3. Male and particularly female gametocytes are likely storing many transcripts for later in the invertebrate developmental stages and thus may not be translated into proteins in this part of the lifecycle in the blood [14, 15].

## Supplementary Information

**Additional file 1: Table S1.** Promoter sequences. **Table S2.** Integration primers. **Table S3.** QPCR primer sets. **Table S4.** QRTPCR.

**Additional file 2: Figure S1.** A The modified p230p locus (illustrated for mCherry) where P corresponds to the promoter region of Dynein Heavy Chain or LCCL domain containing protein, ccp2. Male and female tagged gametocyte lines were generated by transfection with the plasmids PcDHC_230p__mC and PcCCp2_230p__mC. B PCR analysis of genomic DNA in the modified P. chabaudi AS lines showing correct integration into the p230p locus of chromosome 3. **Figure S2.** Representative examples of the gating strategies applied to iRBC samples from the A) PcASEFmC_230p_ (all), B) PcASccp2mC_230p_ pRBC (♀), C) PcASDHCmC_230p_ pRBC (♂) stained and sorted on a BDFACS AriaTM Fusion B flow cytometer, equipped with 375, 561 and 640 nm lasers [mCherry 610/20, Hoechst 450/50, NIR 780/60].

## Data Availability

The datasets generated and analysed during the current study are available on ENA, Project: PRJEB53024. https://www.ebi.ac.uk/ena/browser/view/PRJEB53024?show=reads

## Abbreviations

AAPL: Acute Associated pir Locus
ChAPL: Chronic Associated pir Locus
