# Supplementary material for: Identification of gametocyte-associated pir genes in the rodent malaria parasite, Plasmodium chabaudi chabaudi AS
Source: BMC Res Notes. 2023 Apr 19;16:56. doi: 10.1186/s13104-023-06322-1 (PMC10114299; doi:10.1186/s13104-023-06322-1)
Supplement: Supplementary file 2 — Additional file 2: Figure S1. A The modified p230p locus (illustrated for mCherry) where P corresponds to the promoter region of Dynein Heavy Chain or LCCL domain containing protein, ccp2. Male and female tagged gametocyte lines were generated by transfection with the plasmids PcDHC230p_mC and PcCCp2230p_mC. B PCR analysis of genomic DNA in the modified P. chabaudi AS lines showing correct integration into the p230p locus of chromosome 3. Figure S2. Representative examples of the gating strategies applied to iRBC samples from the A) PcASEFmC230p (all), B) PcASccp2mC230p pRBC (♀), C) PcASDHCmC230p pRBC (♂) stained and sorted on a BDFACS AriaTM Fusion B flow cytometer, equipped with 375, 561 and 640 nm lasers [mCherry 610/20, Hoechst 450/50, NIR 780/60]. [file 13104_2023_6322_MOESM2_ESM.docx]

**Additional File Figure 1**

1. The modified p230p locus (illustrated for mCherry) where P corresponds to the Dynein Heavy Chain or LCCL domain containing protein, ccp2 promoter region. Male and female tagged gametocyte lines were generated by transfection with the plasmids *Pc*DHC_230p__mC and *Pc*CCp2_230p__mC.
2. PCR analysis of genomic DNA in the modified *P. chabaudi* AS lines showing correct integration into the p230p locus of chromosome 3. A) 5’ integration (P1/P3); B) 3’ integration (P4/P2); C) Wild-type locus (P1/P2). Lanes 1-4: *Pc*ASDHCmC_230p_ pRBC ( ♂ ), *Pc*ASccp2mC_230p_ pRBC (♀ ); wild type *Pc*AS pRBC, water control. Primers used are listed in Supplementary Table 1.

Unprocessed original gel image. Top= A) 5’ integration (P1/P3); Middle 1=B) 3’ integration (P4/P2); Middle 2=C) Wild-type locus (P1/P2); Bottom: Irrelevant PCR primer set. Relevant lanes are indicated in the annotated gel image below. L1 (Thermofisher Invitrogen 1Kb Plus; top marker 12kb) and L2 (Hyperladder I, Bioline, top marker 1013 kb), relevant transgenic lines in annotated lanes 1-2, wild-type line in lane 5 and water control in lane 6. All other lanes contain lines which were not used in this study.


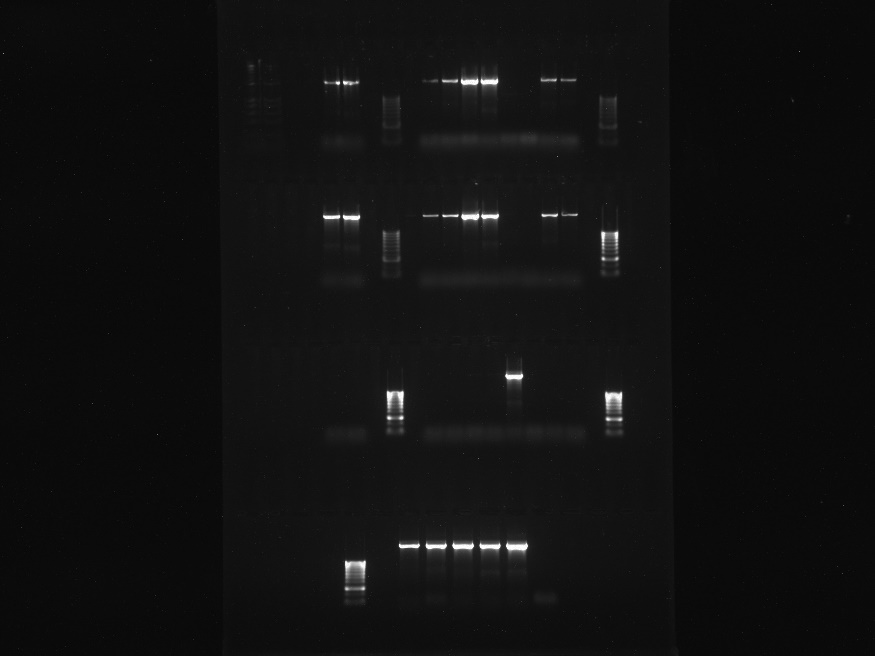


Annotated gel image of the above gel showing additional luciferase tagged lines not used in this study as detailed: PCR analysis of genomic DNA in the modified *P. chabaudi* AS lines showing correct integration into the p230p locus of chromosome 3. A) 5’ integration (P1/P3); B) 3’ integration (P4/P2); C) Wild-type locus (P1/P2). *D) Samples not relevant to this study.* Lanes 1-6: *Pc*ASDHCmC_230p_ pRBC ( ♂ ), *PcASDHCluc_230p_ pRBC ( ♂ ),* *Pc*ASccp2mC_230p_ pRBC (♀ ) *PcASccp2luc_230p_ pRBC (♀ );* wild type *Pc*AS pRBC, water control. Primers used are listed in Supplementary Table 1. *(Italicised lines not used in this study).* Relevant lanes are indicated in the annotated gel image. L1 (Thermofisher Invitrogen 1Kb Plus; top marker 12kb) and L2 (Hyperladder I, Bioline, top marker 1013 kb), relevant transgenic lines in annotated lanes 1-2, wild-type line in lane 5 and water control in lane 6. All other unannotated lanes contain other parasite lines not used in this study.


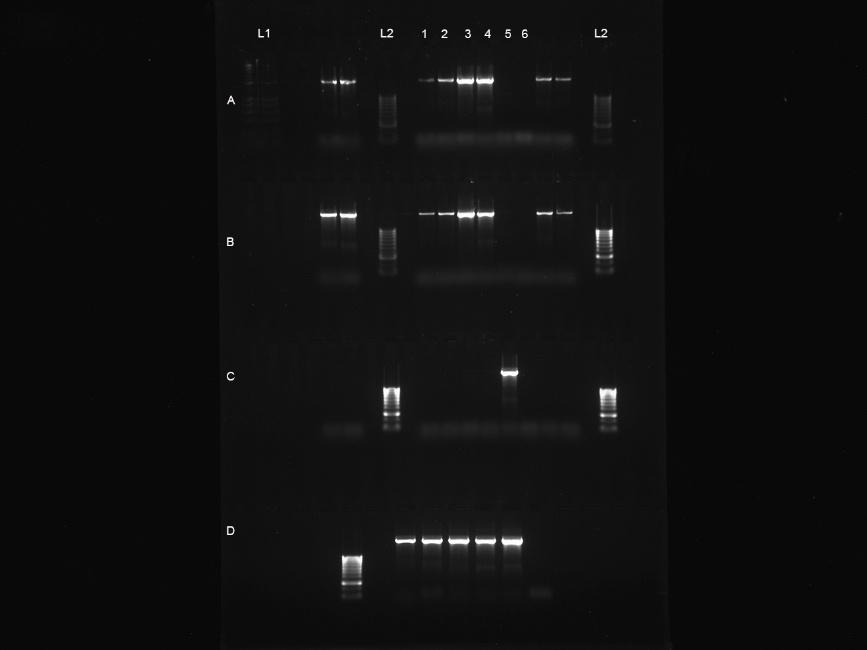


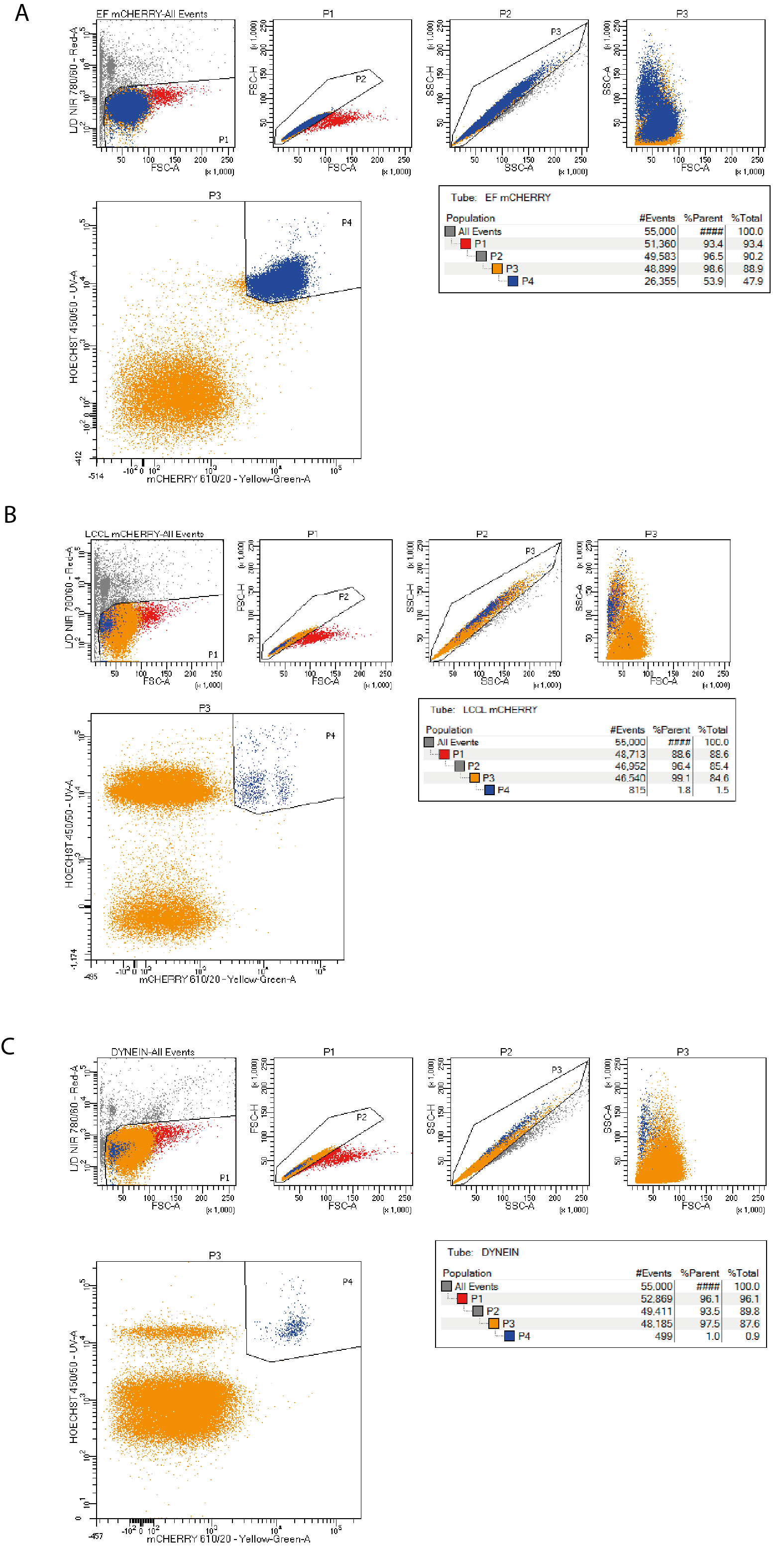


**Additional File 1 Figure 2.**

Representative examples of the gating strategies applied to iRBC samples from the A) *Pc*ASEFmC_230p_  (all), B) *Pc*ASccp2mC_230p_ pRBC (♀ ), C) *Pc*ASDHCmC_230p_ pRBC ( ♂ ) stained with Hoechst 33342 and LIVE/DEAD™ Fixable Near-IR (NIR) Dead Cell Stain and sorted on a BDFACS Aria^TM^ Fusion B flow cytometer, equipped with 375, 561 and 640 nm lasers [mCherry 610/20, Hoechst 450/50, NIR 780/60]. The live Hoechst 33342/mCherry double positive population (P4) was collected directly into Trizol, for RNA analysis or into IMDM supplemented with 2% FCS, for microscopy.
